# Supplementary material for: Specific Inhibition of VanZ-Mediated Resistance to Lipoglycopeptide Antibiotics
Source: Int J Mol Sci. 2021 Dec 22;23(1):97. doi: 10.3390/ijms23010097 (PMC8744867; doi:10.3390/ijms23010097)
Supplement: Supplementary file 1 [file ijms-23-00097-s001.zip › ijms-1498412-supplementary.pdf]

Supplementary data for: Specific inhibition of VanZ-mediated resistance to lipoglycopeptide antibiotics

Vishma Pratap Sur [1,†], Aninda Mazumdar [2,†], Vladimir Vimberg [2,†], Tommaso Stefani [2], Ladislav Androvic [3], Lucie Kracikova [3,4], Richard Laga [3], Zdenek Kamenik [2] and Katerina Komrskova [1,5,*]

1 Laboratory of Reproductive Biology, Institute of Biotechnology of the Czech Academy of Sciences, BIOCEV, Prumyslova 595, 252 50 Vestec, Czech Republic; vishmapratap.sur@ibt.cas.cz
2 Laboratory for Biology of Secondary Metabolism, Institute of Microbiology of the Czech Academy of Sciences, Videnska 1083, 142 20 Prague, Czech Republic; aninda.mazumdar@biomed.cas.cz (A.M.); vladimir.vimberg@gmail.com (V.V.); tommaso.stefani@biomed.cas.cz (T.S.); kamenik@biomed.cas.cz (Z.K.)
3 Department of Polymer and Colloid Immunotherapeutics, Institute of Macromolecular Chemistry of the Czech Academy of Sciences, Heyrovskeho namesti 2, 162 06 Prague, Czech Republic; androvic@imc.cas.cz (L.A.); woldrichova@imc.cas.cz (L.K.), laga@imc.cas.cz (R.L.)
4 Department of Polymers, Faculty of Chemical Technology, University of Chemistry and Technology, Prague, Technicka 5, 166 28 Prague, Czech Republic
5 Department of Zoology, Faculty of Science, Charles University, Vinicna 7, 128 44 Prague, Czech Republic
* Correspondence: katerina.komrskova@ibt.cas.cz
† Authors equally contributed.

1. I-TASSER Results

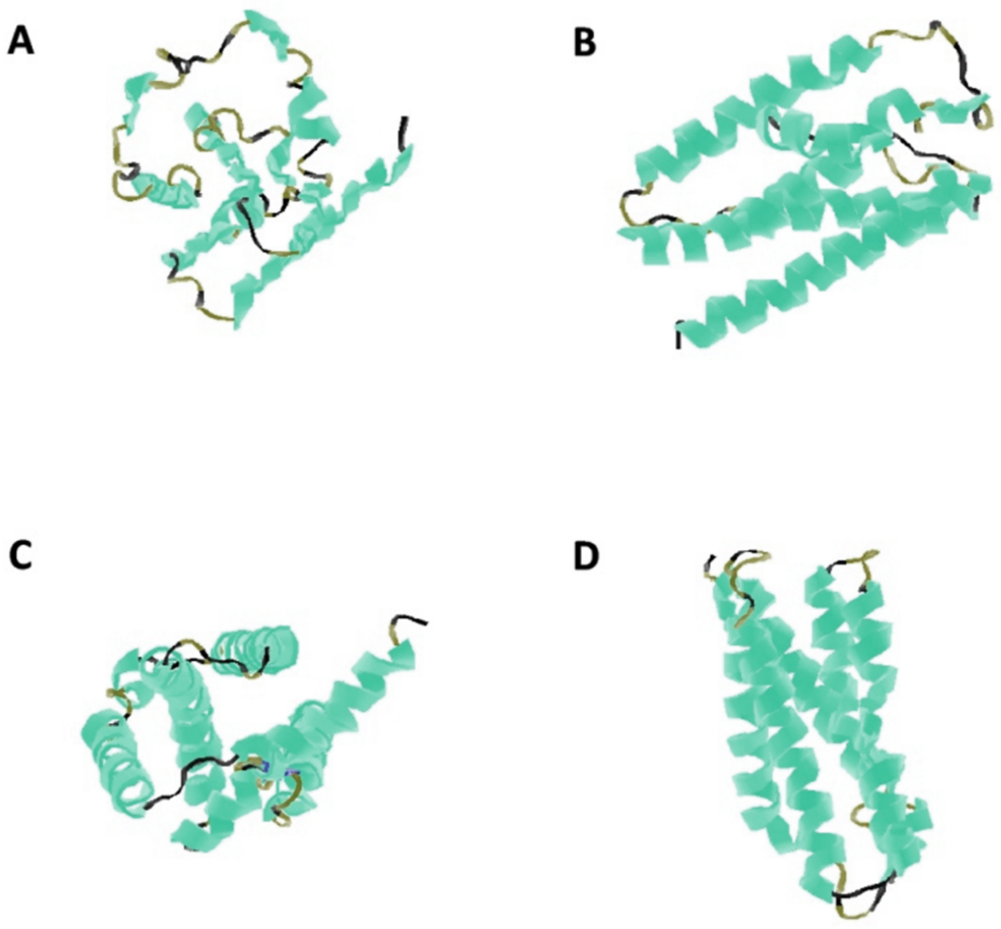

Figure S1. The four other models obtained from I-TASSER for VanZ.

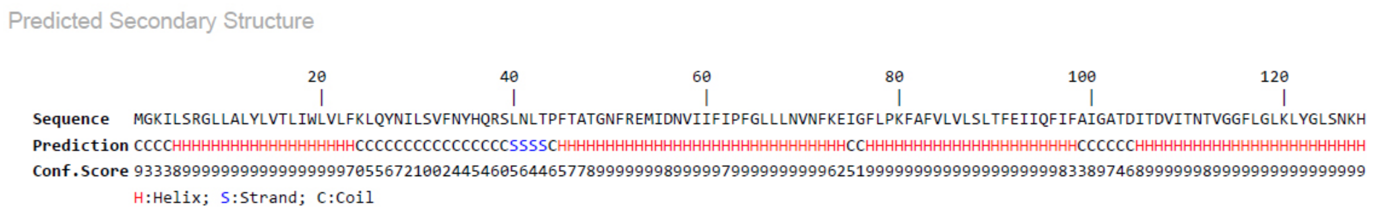

Figure S2. Predicted secondary structure and confidence score.

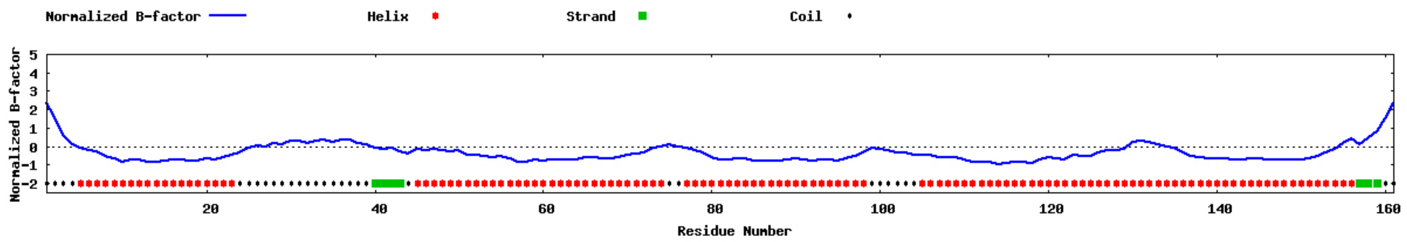

Figure S3. The predicted normalized B-factor value.

Table S1. The ten templates and its Z-score.

Rank	PDB hits	Normalized Z-score
1	6exsA	1.00
2	4ryiA	1.30
3	6y79	1.16
4	6u0oA	1.06
5	3wajA	1.02
6	4o6yA	1.16
7	6rfq	1.16
8	6ob6A	1.04
9	5t4oK	1.15
10	7a23	1.16

Table S2. Models and their C-score.

Model	C-score
1	-2.13
2	-4.72
3	-3.93
4	-4.91
5	-5.00

2. Ramachandran plot

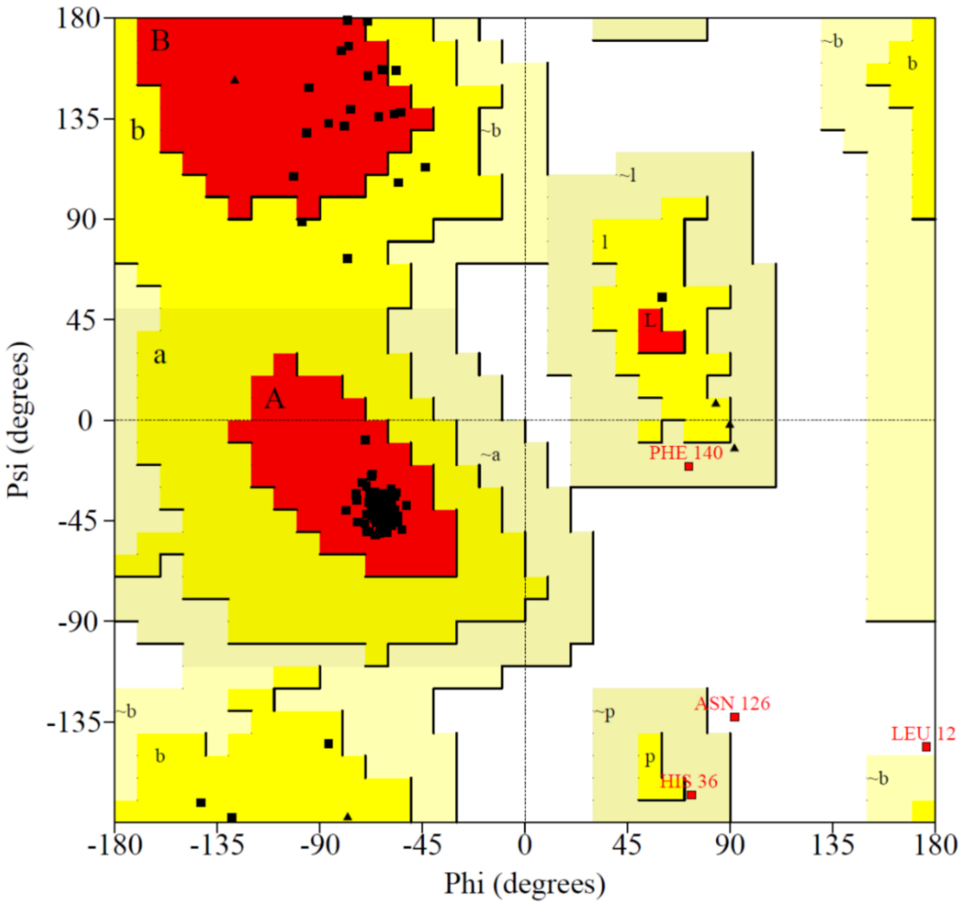

Figure S4. Ramachandran Plot for VanZ

3. Docking results

Table S3. The docking score of all the VanZ-G3K pose.

Ligand	Binding Affinity Kcal/mol
vps_C15H11N7O_uff_E=728.43	-7.7
vps_C15H11N7O_uff_E=728.43	-7.6
vps_C15H11N7O_uff_E=728.43	-7.3
vps_C15H11N7O_uff_E=728.43	-7.2
vps_C15H11N7O_uff_E=728.43	-7.2
vps_C15H11N7O_uff_E=728.43	-7.1
vps_C15H11N7O_uff_E=728.43	-7.0
vps_C15H11N7O_uff_E=728.43	-6.9
vps_C15H11N7O_uff_E=728.43	-6.9

4. Membrane model for understanding the amino acid positioning

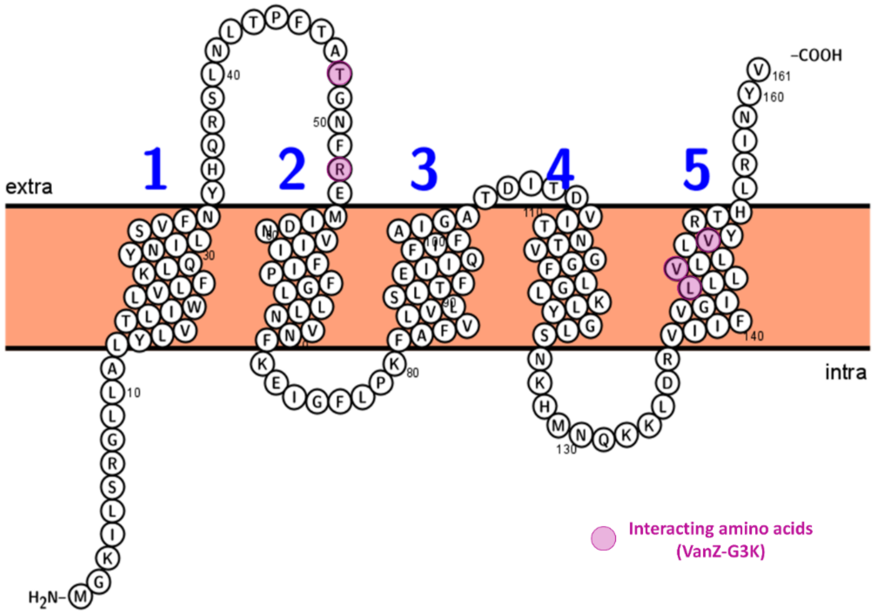

Figure S5. Protter based prediction for transmembrane regions marked with interacting amino acids.

5. Chemical synthesis of G3K

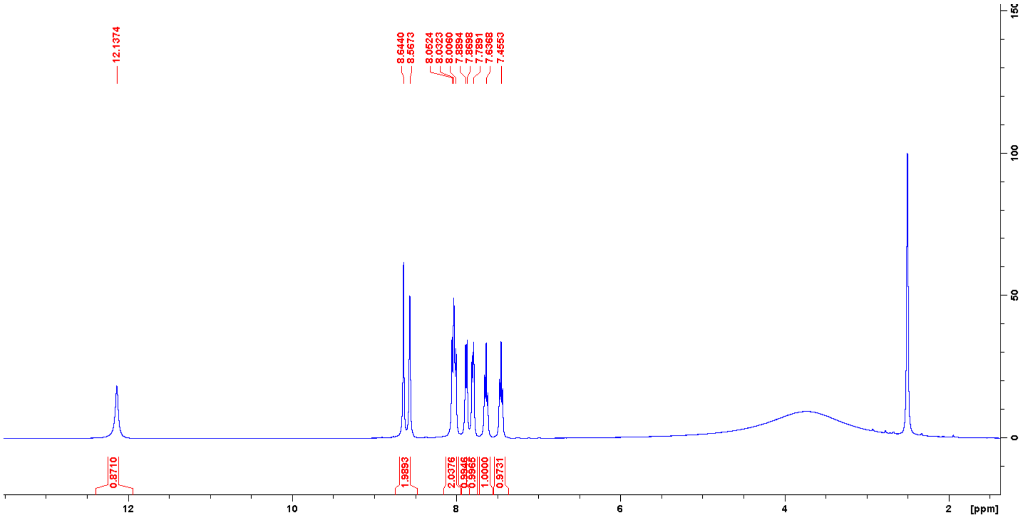

Figure S6. The ^{1}H-NMR spectra of G3K.

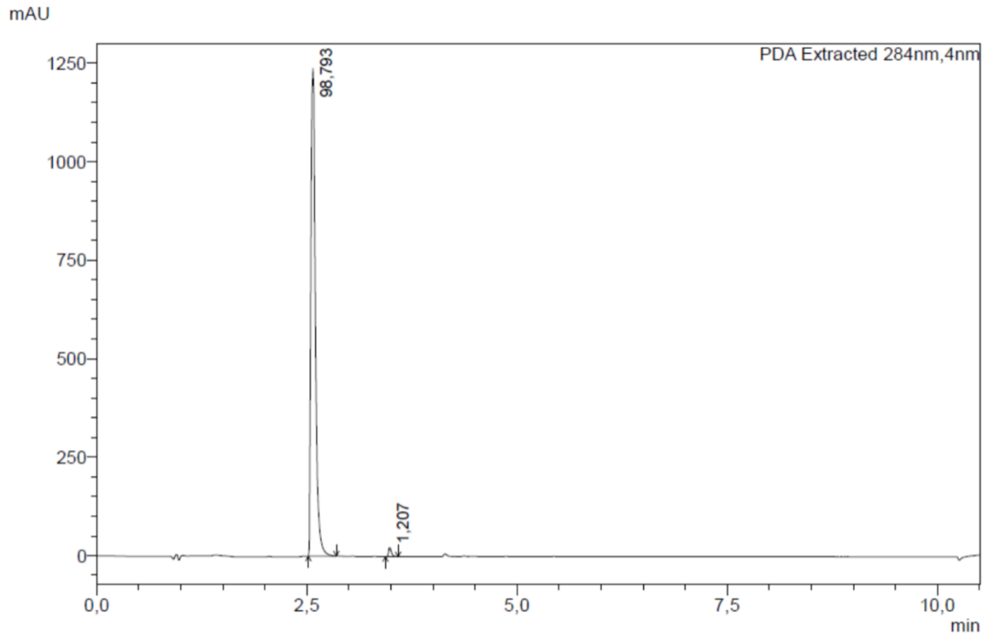

Figure S7. The chromatogram of G3K plotted as the relative response of the PDA detector (AU) at 284 nm over time.

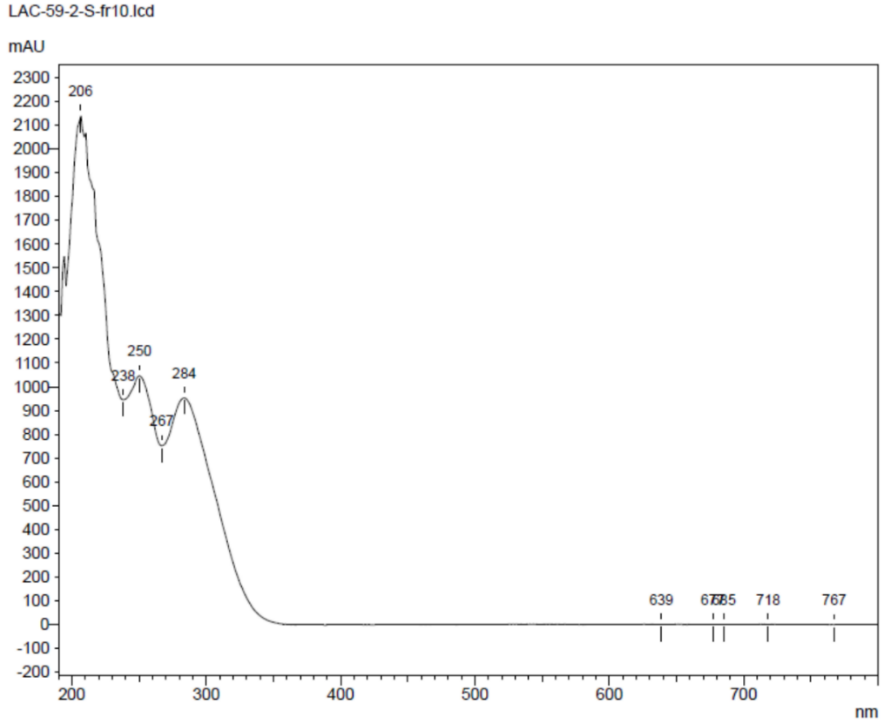

Figure S8. The UV-Vis spectrum of G3K plotted as the dependence of the relative absorption unit (AU) on the wavelength.

6. Antibacterial activity of G3K.

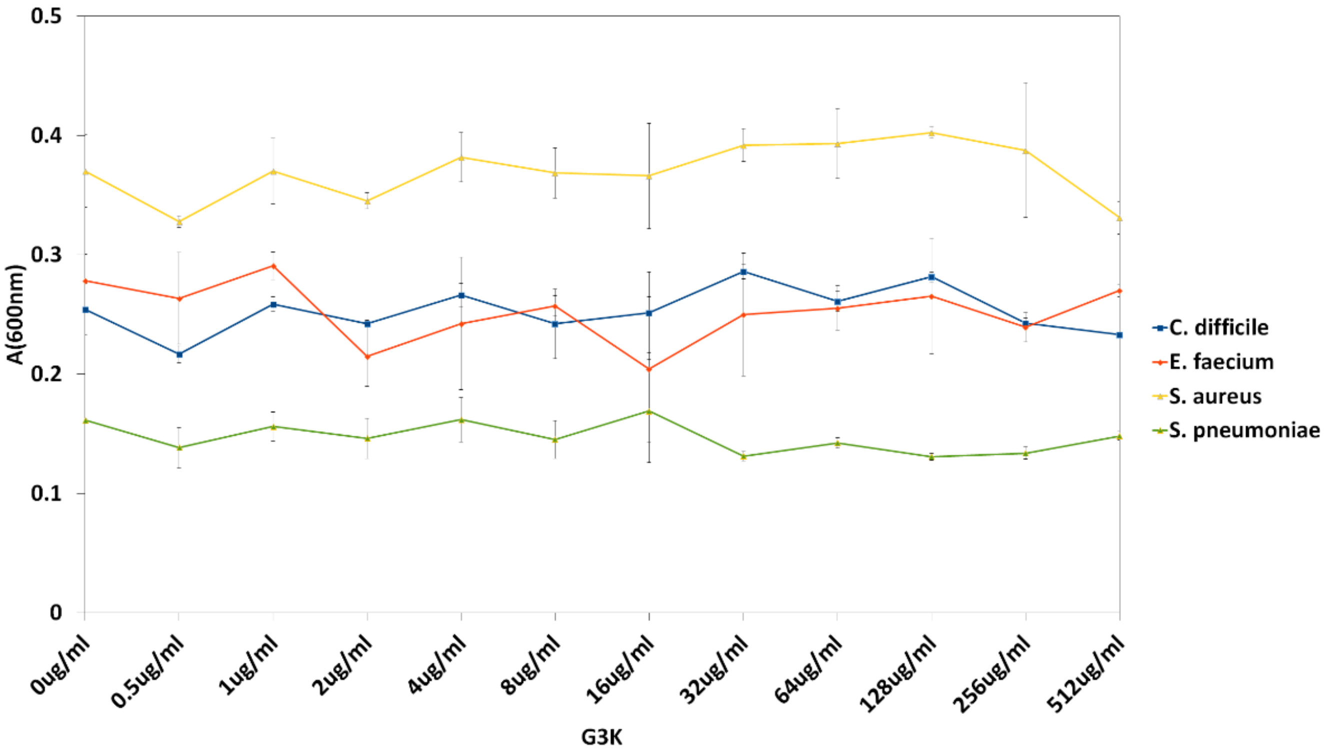

Figure S9. Effect of increasing the amount of G3K on bacteria yield after overnight growth.
